# 重症颅脑创伤后出凝血障碍1例报告并文献复习

**DOI:** 10.3760/cma.j.cn121090-20241208-00545

**Published:** 2024-12

**Authors:** 安俐 刘, 孝媛 董

**Affiliations:** 1 山东第一医科大学附属省立医院老年血液肿瘤科，济南 250021 Department of Geriatric Hematology and Oncology, Shandong Provincial Hospital Affiliated to Shandong First Medical University, Jinan 250021, China; 2 山东大学齐鲁医院血液病研究室，济南 250012 Institute of Hematology, Qilu Hospital of Shandong University, Jinan 250012, China; 3 山东大学齐鲁医院血液科，济南 250012 Department of Hematology, Qilu Hospital of Shandong University, Jinan 250012, China

**Keywords:** 创伤性脑损伤, 创伤后凝血功能障碍, 脑心综合征, Traumatic brain injury, Trauma-induced coagulopathy, Cerebrocardiac syndrome

## Abstract

**目的:**

提高对重症颅脑创伤后出凝血障碍诊断及治疗的认识。

**方法:**

报告重症颅脑创伤后出凝血障碍1例并进行文献复习。

**结果:**

患者女，70岁，因颅脑外伤术后意识不清10小时入院。患者入院后急行硬膜外血肿清除术+去骨瓣减压术+硬膜外血肿引流术，发生凝血障碍，诊断颅脑创伤后出凝血障碍，次日心梗并发心衰、下肢静脉血栓，经多次输注凝血酶原复合物等支持治疗，应用低分子肝素、积极抗感染治疗等，患者最终好转出院。

**结论:**

对于创伤尤其是颅脑外伤患者，需尽早进行凝血检测，采取措施维持凝血功能，早期应用血浆或纤维蛋白原，同时应警惕后期高凝状态和血栓形成，适时抗凝。

## Abstract (en)

**Objective:**

To improve the understanding of the diagnosis and treatment of coagulation disorders after severe head trauma.

**Methods:**

One case of coagulation disorder after severe traumatic brain injury was reported and the literature was reviewed.

**Results:**

A 70-year-old female was admitted to the hospital with 10 hours of unconsciousness after head injury. The patient underwent emergency epidural hematoma removal, decompression, and epidural hematoma drainage, and then coagulation disorder occurred, followed by myocardial infarction with heart failure and venous thrombosis of the lower limbs. After multiple infusions of prothrombin complex, application of low molecular weight heparin and active anti-infective therapy, the patient eventually regained coagulation function.

**Conclusion:**

For trauma patients, especially those with head injuries, it is crucial to test coagulation as early as possible, take measures to maintain coagulation function, apply plasma or fibrinogen in the early stages, and be vigilant for later hypercoagulable states and thrombosis, with timely anticoagulation as needed.

创伤性脑损伤（traumatic brain injury，TBI）是全世界面临的严重健康问题，是创伤死亡及残疾的主要原因。严重的TBI后常常伴随创伤性凝血病（trauma-induced coagulopathy, TIC）的发生，由于大出血和组织损伤激活凝血、纤溶和抗凝途径，出现急性凝血功能紊乱。大量组织因子暴露导致凝血异常激活，引起组织损伤和低灌注状态，涉及到内皮损伤、炎症反应、凝血和抗凝血系统的失衡以及血小板功能的异常[1]。这种复杂的相互作用导致了临床上难以预测和治疗的出血倾向，增加了创伤患者的死亡风险[2]。TBI引起的凝血功能紊乱往往预后不良，其住院死亡率可高达50％。在TIC发展的早期阶段，通常存在创伤诱发止血和纤溶亢进，表现为凝血酶原时间（PT）和活化部分凝血活酶时间（APTT）延长、血小板计数（PLT）和纤维蛋白原（FIB）水平降低等，患者常有出血；后期的TIC则以高凝血状态为特征，与静脉血栓栓塞和多器官衰竭相关[3]–[4]。TIC的治疗需要在控制出血和预防血栓形成之间找到平衡，根据病程阶段的主要矛盾，凝血管理方法包括止血（如氨甲环酸）[5]–[6]、液体复苏、血液制品输注以及抗凝药物治疗等。本文将分享重症颅脑创伤后出凝血障碍1例，并对TBI后凝血异常的治疗进行文献复习。

## 病例资料

患者女，70岁，因颅脑外伤术后意识不清10 h入院。患者10 h前发生车祸，伤后意识不清，伴恶心、呕吐多次血性液体，伴鼻腔及右侧外耳道出血，于外院行颅脑CT检查示硬膜外血肿、脑挫伤，3 h后复查颅脑CT显示颅内血肿量明显增大，中线结构移位，脑疝形成，行硬膜外血肿清除术+去骨瓣减压术。入我院时呈昏迷状态，气管插管辅助呼吸。既往史：高血压病20年，口服“苯磺酸氨氯地平”治疗，血压控制可。查体：体温36.5 °C，心率85次/min，呼吸24次/min，血压145/90 mmHg（1 mmHg=0.133 kPa）。昏迷，刺痛不睁眼，刺痛躲避，格拉斯哥昏迷评分（GCS评分）6分，右额颞顶颅骨缺损，骨窗区压力较高，枕部可见皮下瘀斑，双侧鼻腔及右侧外耳道可见新鲜血液，颈部明显抵抗感，双肺广泛痰鸣音。心、腹部（−）。头部硬膜外引流管1根，可见血性液体引出，对光反射未见明显异常。凝血指标：PT 18.2 s，FIB 1.08 g/L，国际标准化比率（INR）1.48，凝血酶时间（TT）18.2 s，D-二聚体4.02 µg/ml，纤维蛋白原降解产物（FDP）18.56 µg/ml；血常规：HGB 57 g/L，PLT 83×10^9^/L；血生化：K^+^ 3.28 mmol/L，Cl^-^ 115 mmol/L，Ca^2+^ 1.58 mmol/L；血气：pH 7.42，HCO_3_^-^ 25.3 mmol/L，TCO_2_ 26.5 mmol/L，碱剩余（BE）0.8 mmol/L，PCO_2_ 37 mmHg；生化：白蛋白16 g/L；心肌损伤标志物：心肌肌钙蛋白I（CTNI）53.89 ng/ml，肌酸激酶同工酶（CK-MB）5 mg/L，氨基末端脑利钠肽前体（NT-proBNP）450 ng/L；血栓弹力图：反应时间（R）7.5 min，凝固时间（K）5.8 min，α角（Angle）37.7°，最大振幅（MA）42.3 mm，血块溶解速率预测值（EPL）0，血凝块减少速率（LY30）0，凝血综合指数（CI）−6.8，血小板最大振幅（PMA）1.0 mm，指示低凝，纤维蛋白原功能低，血小板功能低。心电图：心内膜下层心梗，Ⅱ、V3、V4、V5、V6 ST-T改变。入院后复查颅脑CT：右侧硬膜外血肿、硬膜下血肿，多发脑挫伤、蛛网膜下腔出血、脑室内积血，中线结构基本居中。双肺炎症、纤维灶，双侧胸膜积液。考虑诊断：1. 重度颅脑外伤术后（脑疝术后、右侧硬膜外血肿、右侧硬膜下血肿、双侧多发脑挫裂伤、蛛网膜下腔出血、脑室内积血、颅底骨折、脑脊液鼻漏、脑脊液耳漏、颅骨骨折、头皮血肿）；2. 创伤后凝血障碍；3. 肺部感染；4. 创伤性湿肺；5. 双侧胸腔积液；6. 高血压病（2级，中危）。

入院当日行硬膜外血肿引流术，颅内注射尿激酶。术后给予凝血酶原复合物1 200 U、纤维蛋白原3 g、血浆600 ml、红细胞14 U、白蛋白，甘露醇降颅压，哌拉西林他唑巴坦抗感染。次日复查WBC 12.54×10^9^/L，HGB 83 g/L，PLT 67×10^9^/L；凝血基本正常；CTNI 4 218.32 ng/ml，CK-MB 37.8 mg/L，NT-proBNP 1 257 ng/L，考虑心梗并心力衰竭，给予酒石酸美托洛尔、重组人脑利钠肽、托拉塞米、缬沙坦氨氯地平、乌拉地尔。d3 B超示左下肢肌间静脉血栓。d4 PT 14.6 s，APTT 25.2 s，FIB 1.17 g/L，D-二聚体48.51 µg/ml；CTNI 53.89 ng/ml，CK-MB 5 mg/L，NT-proBNP 450 ng/L。脑脊液涂片见少量革兰阴性杆菌（G^-^菌），球蛋白+，Cl^-^ 162 mmol/L。多次腰椎穿刺，促进血性脑脊液循环，并腰大池置管引流。给予派拉西林他唑巴坦抗感染。d5颅脑新出现右侧基底节低密度，梗死灶可能大。d6 WBC 9.16×10^9^/L，HGB 100 g/L，PLT 81×10^9^/L；PT 19.6 s，APTT 44.7 s，FIB 0.6 g/L，TT 23.7 s，D-二聚体75.95 µg/ml，FDP 88 µg/ml；脂肪酶1 398 U/L，淀粉酶196 U/L。胃液潜血试验（OB）+。凝血酶原复合物300 U、纤维蛋白原2 g、血浆、冷沉淀、血小板、红细胞，转入ICU。随后2 d分别给予纤维蛋白原2 g和4 g。凝血改善。d7患者发热，体温38.3 °C，口腔内黄色伴血性黏痰，WBC 14.0×10^9^/L，ANC 12.17×10^9^/L，痰培养：嗜麦芽窄食单胞菌，多种抗生素敏感。给予物理降温，派拉西林他唑巴坦改为头孢哌酮舒巴坦。d10复查WBC 16.29×10^9^/L，ANC 14.84×10^9^/L，降钙素原（PCT）1.57 ng/ml，凝血无异常。此时应用低分子肝素预防血栓，头孢哌酮舒巴坦升级为美罗培南。d11患者发热38.9 °C，枕后大面积皮下瘀斑，D-二聚体12.15 µg/ml，大便少量真菌孢子，心肌损伤标志物较前好转。超声示双下肢肌间静脉血栓形成，左侧胫后、腘静脉血栓形成。d12患者夜间高热39 °C，出现双下肢花斑，PCT 0.785 ng/ml，尿白细胞、红细胞、蛋白均阳性。低分子肝素改为依诺肝素，加用万古霉素。d13患者仍发热，血培养见鲍曼不动杆菌，C-反应蛋白（CRP）18.4 mg/L，大便球杆菌比例失调，痰涂片见G^-^菌。拔除腰大池引流，加用利奈唑胺。d17患者神志间断好转，未再发热。复查CT：出血较前改善，仍有右侧基底节低密度灶，双肺炎症较前明显好转，余变化不大。d18患者未再发热，脑脊液提示颅内感染可能性小。血栓弹力图：R 75.5 min，K 1.2 min，Angle 72.5°，MA 76.8 mm，EPL 0，LY30 0，CI 3.1，PMA 0，高凝，FIB功能高，血小板功能高。停用美罗培南，改为哌拉西林他唑巴坦+利奈唑胺+万古霉素治疗。d22患者体温正常，WBC 10.01×10^9^/L，HGB 97.0 g/L，PLT 313×10^9^/L，PCT（−），大便球杆菌好转，停派拉西林、利奈唑胺，仅万古霉素维持抗感染。d27患者低热37.3°C，尿中有絮状物，尿培养见铜绿假单胞菌，WBC 11.38×10^9^/L，ANC 8.28×10^9^/L，CRP、PCT（−），复查CT：出血较前改善，仍有右侧基底节低密度灶，余变化不大。停用万古霉素，改为派拉西林抗感染，氟康唑膀胱冲洗。d31患者未再发热，WBC 7.79×10^9^/L，尿培养未见细菌真菌，停派拉西林。d33超声：双下肢肌间静脉血栓，未提示胫后、腘静脉血栓。患者意识模糊，存在认知障碍，混合性失语，可经口进食，血常规、凝血正常，感染指标阴性。给予床旁肢体功能、认知及语言等康复训练及针灸治疗，拔除胃管。d39患者可床上适当肢体活动，但仍存在认知功能障碍，大小便失禁，时有幻觉发生，可能存在器质性行为障碍，出院。

## 讨论及文献复习

正常人的出凝血稳定是建立在出血和血栓形成之间的微妙平衡上，当发生颅脑外伤，这种平衡很容易被打破。凝血功能障碍是影响TBI患者临床病程的常见原因，近三分之二的患者在入院常规检查时发现凝血功能异常。凝血功能障碍包括低凝状态（出血）和高凝状态（血栓形成），两者可在脑外伤后同时发生。通常认为TBI患者凝血障碍的临床过程和出血增多是由于高凝状态向低凝状态的快速进展，受损脑组织首先释放促凝血的组织因子，然后高凝状态下凝血因子不断地被消耗，血小板功能紊乱、内源性抗凝作用、血管内皮细胞激活、纤维蛋白原变性、炎症反应和纤溶亢进，这些引起凝血障碍的病理生理学机制都会引起增加再出血的风险[7]。在不同研究中，TBI患者的TIC发生率差异很大（7％～97.5％），当TBI合并不同程度的凝血功能障碍，其死亡率也有很大差异（17％～86％）[8]，且发生TIC的患者病死率5倍于未发生TIC的患者[9]。研究表明，创伤引起的组织损伤和组织灌注不足是TIC发生的独立危险因素。通过对77例创伤患者的临床资料进行回顾性分析，发现创伤后早期有28％的患者存在PT延长，8％的患者存在APTT延长，这表明TIC可以在创伤后早期发生，并且是创伤后患者发生失血的主要原因之一，也是导致创伤患者病死率增高的原因之一[10]–[11]。

TBI引发的凝血障碍涉及多种病理机制，包括内源性肝素样物质的产生、蛋白C途径的激活、纤维蛋白溶解过程的加剧、血小板功能的失调、体温过低和代谢性酸中毒等。对于TIC患者，失血性休克的发生和血管内皮细胞的广泛损伤引发的炎症因子释放等也会加剧TBI患者的凝血功能异常[12]。根据GCS评分可以将TBI严重程度分为轻度（13～15分）、中度（9～12分）或重度（≤8分）。在TBI患者群体中，血小板功能的减退与较低的GCS评分有关联，且血小板功能障碍的TBI患者病死率10倍于血小板功能正常的患者[13]。TIC的发展可能与创伤后血小板功能紊乱有直接联系。在创伤发生后的早期，患者血小板数量下降，骨髓加速造血，可能使得血小板质量异常，功能下降，并引发或加剧凝血功能异常。外伤后出现以下症状时高度提示TIC：在严重创伤后原因不明的出血或凝血功能异常；补充血容量后，失血性休克的症状仅暂时缓解，迅速复发；PT和APTT延长，凝血因子活性下降；血小板功能降低；纤维蛋白溶解的相关指标异常；体温下降和酸中毒[14]。

研究表明30％的患者会在TBI后发生继发性心肌损伤，早期表现为血流动力学不稳定、心电图异常、心肌损伤标志物的升高以及心功能下降，称为脑心综合征（cardio-cerebral syndrome, CCS）。急性脑损伤引起脑水肿、颅内压升高，体内儿茶酚胺、肾上腺素、血管紧张素Ⅱ、血浆内皮素等水平升高，强烈收缩血管，甚至对心肌产生直接毒性作用，损伤后应激反应导致交感-肾上腺髓质、下丘脑-垂体-肾上腺皮质、肾素-血管紧张素轴分泌增加，引起机体高代谢、高氧耗状态，心脏会出现反应性缺血缺氧，颅脑外伤后的高凝状态也会增加冠脉栓塞风险[15]。颅脑外伤程度越重，心肌损伤发生越早，心肌损伤会导致患者的心脏射血分数、血压降低，脑灌注不足，发生脑缺血，进而继发性脑损伤，甚至休克，TBI后心肌损伤患者6个月内存在意识行为障碍的发生率更高[16]。外周灌注不足会导致组织缺血缺氧，血管内皮细胞损伤严重，并且释放t-PA等物质，加重TBI患者凝血功能障碍[17]。低血压作为最早出现的损伤，与不良预后密切相关[18]，因此维持正常血压是TBI的重要治疗目标，等渗晶体是首选，应避免以林格液为代表的低渗晶体溶液，以减少液体转移到损伤的脑组织。El-Menyar等[19]的研究发现，早期应用β受体阻滞剂可以显著改善TBI引起的心肌损伤标志物及炎性指标升高。因此TBI治疗过程中需要积极处理原发病，纠正失血性休克、凝血异常及水电解质紊乱，及时应用β受体阻滞剂及保护心肌药物。

TBI本身被认为是深静脉血栓的一个独立危险因素[20]，在TBI数天后可能才出现高凝状态[21]。创伤后期的炎症可加重高凝状态，加上前期为逆转低凝状态应用的止血措施，增加了深静脉血栓形成（deep venous thrombosis，DVT）及心梗等血栓栓塞并发症的风险。在缺乏监测和预防的情况下，DVT的发生率很高，在重度TBI患者中高达54％[22]。在TBI之后，不合时宜地预防DVT可能导致颅内出血加重[23]。因此，在TBI后预防血栓形成用药选择、时机仍有相当多的临床不确定性。对长期卧床患者，可以使用各种类型的外部压缩设备来预防DVT，例如弹力袜、间歇充气加压装置和踝泵。使用物理预防后DVT发生率为31％，肺栓塞发生率为3％[24]。大多数的研究表明低分子肝素预防可降低DVT的发生率，但也有研究提示肝素无效[25]。在TBI患者中，不使用或使用普通肝素/低分子肝素预防DVT都存在明确的风险，因此根据风险因素将TBI患者分为高风险和低风险组进行个体化治疗，可以一定程度上降低DVT及出血的发生率[26]。将肝素预防性治疗推迟到伤后24 h，并限于出血风险较低的患者身上，这样再出血的风险与未给予肝素抗凝治疗的患者相似[27]。如果患者近期有使用抗凝剂或抗血小板治疗、凝血功能差、活动性出血或头颅CT提示出血性病变，那么要限制肝素的使用。在头颅CT提示出血性病变的患者中，如果反复的神经影像学未出现血肿进展的证据，在24～72 h开始预防则不会增加再出血的风险，在明确显示病变进展的患者中，可以推迟预防血栓治疗，因为再出血的风险明显大于发生血栓的风险[28]。回顾性研究通过反复CT检查发现，65岁以上的患者在抗凝治疗后有更高的再出血风险[29]。

患者发生TBI后肺部感染发生率为30％[30]，TBI可以改变全身免疫反应甚至造成免疫抑制状态，从而导致感染易感性增加，中重度TBI后大多数患者需要住院接受治疗，通常需要在ICU中度过一段时间，从几天到几个月不等，在此期间患者发生医院获得性感染的风险大大增加[31]。TBI术后发生感染受多种因素影响，因此，在患者入院后应对其进行综合评估和分析，对于高危人群应尽早采取措施，以降低TBI术后肺部感染的发生率。

TBI要积极清除血肿、有效止血，严重创伤患者大量失血，引起失血性休克为TIC发生因素之一，因此积极、有效地止血为阻止进一步出血及病情加重的重要措施之一。严重创伤所致出血可以局部使用止血胶或纤维蛋白原。对于意识状态快速变差和颅内血肿持续增大的TBI患者，开颅手术有助于快速降低颅压，但开颅手术是否能改善患者远期预后，并未达成共识[32]。围手术期的凝血管理尤为重要，包括止血药物的使用、液体复苏的选择、替代治疗和血压管理等综合性问题。对于轻中型TBI患者，早期使用氨甲环酸可以通过抑制纤溶酶的作用，一定程度上减缓颅内出血的进展，氨甲环酸对于重型TBI患者的改善并不明确，国际血栓与止血委员会推荐在TIC患者创伤后早期应用氨甲环酸[33]。异常的凝血过程中会丢失大量凝血因子及血小板等凝血物质，若输注大量晶体液或胶体液扩充血容量，会稀释外周血而加重凝血功能障碍。对于严重出血的患者，常规的治疗方法是在输注红细胞的同时，使用晶体液或胶体液来扩充血容量，只有在患者出现微血管出血的情况下，才会考虑输注新鲜冰冻血浆和血小板[34]。Balvers等[35]进行的多中心研究显示，增加血浆制品输注中红细胞的比例，并合并使用抗纤维蛋白溶解药物，能够减少TIC患者的输血需求，同时降低患者的病死率。对于存在活动性出血的TBI患者，尤其是失血性休克为主要问题，应持续进行限制性容量复苏；如创伤性脑损伤为主要问题，则进行相对宽松的限制性容量复苏以维持脑血流灌注[36]。

TBI后凝血障碍作为导致创伤患者死亡的主要原因之一，与患者预后密切相关，其主要凝血病理特点为低凝血状态与纤维蛋白溶解亢进。本例患者诊疗过程提示，TBI患者需及时识别凝血障碍、心肌损伤、血栓及感染的发生，并进行积极有效的治疗，则可以有效降低TBI患者的病死率。
